# The mental health of health care workers in Oman during the COVID-19 pandemic

**DOI:** 10.1177/0020764020939596

**Published:** 2020-07-08

**Authors:** Abdallah Badahdah, Faryal Khamis, Nawal Al Mahyijari, Marwa Al Balushi, Hashil Al Hatmi, Issa Al Salmi, Zakariya Albulushi, Jaleela Al Noomani

**Affiliations:** 1 South Dakota State University, Brookings, SD, USA; 2 Royal Hospital, Muscat, Oman

**Keywords:** Stress, anxiety, well-being, COVID-19, health care workers

## Abstract

**Background:**

COVID-19 disease is one of the most destructive events that humanity has witnessed in the 21st century. It has impacted all aspects of life and all segments of populations, including already vulnerable health care providers.

**Aims:**

This study sought to detect the prevalence of mental health issues in sample of physicians and nurses working in several health facilities in Oman.

**Method:**

We gauged the mental health conditions of 509 physicians (38.1%) and nurses (61.9 %) using the Perceived Stress Scale, Generalized Anxiety Disorder Scale and World Health Organization Well-Being Index.

**Results:**

The study revealed a high prevalence of stress, anxiety and poor psychological well-being, especially among females, young health care workers and those who interacted with known or suspected COVID-19 patients.

**Conclusion:**

The outcomes of this study support the handful of studies published during this global health crisis that have found that the mental health of health care workers has been harshly affected and predicted that it will continue, to various degrees, to be affected in the foreseeable future. The results of this study highlight the urgency of providing administrative and psychological support as well as current and accurate information on COVID-19 to health care workers.

## Introduction

The severe acute respiratory syndrome coronavirus 2 (SARS-CoV-2), the virus that causes COVID-19 disease, has caused incredible destruction to individuals, institutions and states. It has and will have substantial economic, social and psychological impacts. Health, however, became the crucial concern that eclipses all other matters (Van den Broucke, 2020). Examples of observed and anticipated consequences of the COVID-19 pandemic include stress, feelings of helplessness, anxiety, loneliness and depression. Researchers warn of increasing rates of social problems such as domestic violence, suicide, and alcohol and substance abuse (Clay & Parker, 2020; Reger et al., 2020). A study of American adults found that one in four (25%) were very worried about infection (Wolf et al., 2020). Kaiser Family Foundation (2020) found in national survey that almost half (47%) of US individuals who were sheltering in place reported higher rates of mental health problems due to worry and stress. Of the 47%, 27% said the impact was significant. The same survey found that half of households with health care workers (HCWs) reported COVID-19-related worry and stress. Wang et al. (2020) found that 12.1% of people in China reported poor sleep, 6.3% suffered from anxiety and 17.1% experienced depression during the crisis.

HCWs’ frequent contact with COVID-19 patients, sometimes without proper personal protective equipment, is a profound source of fear, stress, sleep disturbance and anxiety. Despite HCWs’ resilience a substantial number of them have experienced, and will experience, some physical and psychological difficulties that surpassed their capacity to manage them during the current outbreak (Greenberg et al., 2020; Matheson et al., 2016). The prevalence of mental health problems has been documented in several studies (Ahmed et al., 2020; W. Lu et al., 2020). In a study of frontline HCWs in China, Lai et al. (2020) found that 50% had depression, 45% had anxiety and 34% had insomnia. Similar findings were reported during other epidemics. For example, a study during the 2003 SARS outbreak from Taiwan found that 17.3% of HCWs who had interacted with confirmed or suspected SARS patients suffered from mental health problems (Y. C. Lu et al., 2006).

Although COVID-19 is a source of stress, anxiety and anguish for everyone, its impact is significantly tougher for HCWs, especially those who interact with COVID-19 patients. In this study we studied the prevalence of anxiety, stress and well-being and the relationship among these variables among HCWs in Oman, an Arab country in the Arabian Peninsula.

## Materials and methods

We conducted a cross-sectional web-based survey of HCWs during the first 2 weeks of April 2020. The investigation was approved by the Royal Hospital Research Ethics Committee in Oman (SRC# 34/2020). The participants were recruited from 10 multiple health care facilities in Oman. The link of the questionnaire was sent to all potential participants through WhatsApp. All participants were provided with information about the purpose of the study and were assured confidentiality. Participants who consented were permitted to participate in the study.

## Participants

The sample (Table 1) consisted of 315 nurses (61.9%) and 194 physicians (38.1%). The gender distribution was 80.3% females and 19.7% males. The mean age of the participants was 37.67 years (*SD* = 7.68). Most of the HCWs were married (79.5%); the remainder were either single (16.1%) or divorced or widowed (4.3%). About one-quarter (28.5%) of the HCWs worked with patients with known or suspected COVID-19.

**Table 1.** Demographic characteristics of HCWs.

Characteristics	*n* (%)
Full sample^a^	509
Occupation	
Physicians	194 (38.1)
Nurses	315 (61.9)
Gender	
Females	407 (80.3)
Males	100 (19.7)
Contact with COVID patients	
Yes	144 (28.5)
No	361 (70.5)
Marital status	
Married	404 (79.5)
Unmarried^b^	104 (20.4)

*n*: number; *SD*: standard deviation.

a The total might not tally because of missing data. ^b^The unmarried category includes never married, divorced and widowed.

## Measures

We first collected information about age, gender, occupation and contact with COVID-19 patients. Then the participants filled out three standardized composite measures that assessed generalized anxiety disorder, stress and subjective psychological well-being.

### Generalized Anxiety Disorder Scale

The Generalized Anxiety Disorder Scale (GAD-7) is a 7-item retrospective self-report screening tool for generalized anxiety disorder and one of the most commonly used scales to gauge anxiety in both research and clinical settings (Beard & Björgvinsson, 2014; Toussaint et al., 2020). It asks how frequently participants have suffered from several symptoms of anxiety in the previous 2 weeks. Examples of the items include ‘Feeling nervous, anxious, or on edge’ and ‘Feeling afraid, as if something awful might happen’. All items are rated on a 4-point Likert-type scale ranging from 0 (*not at all*) to 3 (*nearly every day*). Total scores range from 0 to 21, with higher scores indicating greater anxiety. The total scores are often categorized into four levels of severity: (1) minimal (0–4), (2) mild (5–9), (3) moderate (10–14) and (4) severe (15–21). A score of 10 or higher signifies a higher degree of anxiety (Spitzer et al., 2006). Cronbach’s alpha for the GAD-7 in this study was .89.

### Perceived Stress Scale

The Perceived Stress Scale (PSS-10) is a 10-item retrospective global measure of stress constructed to measure the degree to which life events are judged as stressful and respondents’ reaction to them (Cohen et al., 1983). The scale consists of a mix of negatively and positively worded items. Examples of the items include ‘How often have you felt nervous or stressed?’ and ‘How often have you felt confident about your ability to handle your personal problems?’ All items are rated on a 5-point Likert-type scale ranging from 0 (*never*) to 4 (*very often*). The total possible score ranges from 0 to 40, with a higher score indicating a higher degree of perceived stress. The PSS-10 is not a diagnostic tool and does not have a cutoff point. Cronbach’s alpha reliability of the PSS-10 in this study was .80.

### World Health Organization Perceived Well-Being Index

The World Health Organization Perceived Well-Being Index (WHO-5) is a retrospective self-report scale that gauges overall subjective psychological well-being. It consists of five positively worded items that require participants to rate their state of well-being during the preceding 2 weeks (e.g. ‘Over the last 2 weeks I have felt cheerful and in good spirits’). The items are rated on a 6-point Likert-type scale from 0 (*none of the time*) to 5 (*all the time*). The scores are transformed into percentage values by multiplying them by 4. The new transformed scores range from 0 (*worst possible well-being*) to 100 (*best possible well-being*). Several studies indicated that a score of less than 50 (or a raw score ⩽ 12) indicates diminished well-being and a score <28 is an indicator of likely depression (Cichoń et al., 2020; Halliday et al., 2017). Cronbach’s alpha for internal consistency for the WHO-5 was .90

## Results

The scores on the GAD-7 showed that the largest proportion of HCWs (74.1%) experienced minimal to mild anxiety (35.5% and 38.7%, respectively). The rest (25.9%) reported moderate to severe anxiety (17.7% and 8.3%, respectively). A chi-square analysis (Table 2) revealed that a significantly greater proportion of females than males scored 10 or higher (χ^2^ (1) = 5.28, *p* = .01). The same test revealed no significant differences in anxiety levels between nurses and doctors (χ^2^ (1) = .48, *p* = .28), nor between HCWs who cared for COVID-19 patients and those who did not (χ^2^ (1) = 1.45, *p* = .14). Also, there was no significant difference in the proportion of married and nonmarried participants who scored 10 or higher on the GAD-7 (χ^2^ (1) = .25, *p* = .35). A weak but significant negative relationship was observed between age and anxiety (*r* = −.12, *p* = .02).

**Table 2.** Prevalence of anxiety, stress and well-being, by gender.

Variables	Gendera			χ^2^	*p* value
	Total	Male	Female		
	509	100	407		
	*n* (%)	*n* (%)	*n* (%)		
GAD-7^b^					
Low anxiety	377 (74.1)	83 (83)	292 (71.7)	5.28	.013
High anxiety	132 (25.9)	17 (17)	115 (28.3)		
PSS-10^c^					
Low stress	222 (43.6)	53 (53)	186 (41.3)	4.49	.023
High stress	287 (56.4)	47 (47)	239 (58.7)		
WHO-5^d^					
Low well-being	220 (43.4)	35 (35)	185 (45.5)	3.57	.037
High well-being	287 (56.6)	65 (65)	222 (54.5)		

*n*: number; GAD: Generalized Anxiety Disorder; WHO-5: WHO Well-Being Index; PSS-10: Perceived Stress Scale. *N* = 509.

a Total might not tally because of missing cases. ^b^ High on GAD defined as a score of 10 or higher.

c High on PSS-10 defined as a score of 24 or higher. ^d^ High on WHO-5 defined as a score of 55 or higher.

The mean score on the PSS-10 was 24.19 (*SD* = 5.84). We used the mean score of the sample as the cutoff value to distinguish between low stress (<24) and high stress (⩾24). A chi-square analysis revealed that a significantly higher percentage of females scored ⩾24 than males (χ^2^ (1) = 4.49, *p* = .02). Similarly, a significantly higher proportion of HCWs who worked closely with COVID-19 patients reported a high level of stress than those who did not (χ^2^ (1) = 4.79, *p* = .02). Neither occupation (physicians vs nurses; χ^2^ (1) = 1.85, *p* = .10) nor marital status (married vs nonmarried) were related to PSS-10 scores (χ^2^ (1) = 2.80, *p* = .06). The age of the HCWs was significantly negatively correlated with PSS-10 scores (*r* = −.29, *p* = .00).

The mean of the transformed WHO-5 was 54.61 (*SD* = 23.09). Female HCWs experienced lower psychological well-being than males (*t* (505) = 2.75, *p* = .01; Cohen’s *d* = .31). No difference was observed between physicians and nurses (*t* (507) = 1.64, *p* = .10), nor between married and nonmarried participants in their overall level of psychological wellness (*t* (503) = 1.11, *p* = .27). HCWs who cared for COVID-19 patients experienced a lower level of well-being than those who did not (*t* (503) = 2.61, *p* = .01, Cohen’s *d* = .65). Pearson correlation analysis revealed that older HCWs experienced more positive well-being than younger ones (*r* = .17, *p* = .00). Using a cutoff of <28, a chi-square analysis revealed that a significantly higher proportion of females scored less than 28 males (χ^2^ (1) = 3.14, *p* = .05). Similarly, a significantly higher proportion of HCWs who did not work with COVID-19 patients scored higher than 28 compared to those who did (χ^2^ (1) = 3.27, *p* = .05). The same test revealed no significant differences in the proportion of nurses and doctors (χ^2^ (1) = .68, *p* = .24), nor in the proportion of married and nonmarried who scored <28 on WHO-5 (χ^2^ (1) = 1.94, *p* = .11).

Pearson correlations between the three measures were significant and in the expected directions. Specifically, significant negative correlations were detected between the WHO-5 and PSS-10 (*r* = −.59, *p* = .00) and the WHO-5 and GAD-7 (*r* = −.59, *p* = .00), while the PSS-10 and GAD-7 were significantly positively correlated (*r* = .55, *p* = .00).

## Discussion

The findings revealed a pessimistic portrait of the mental health of HCWs in Oman. Based on the GAD-7, one in four (26%) HCWs suffered either from moderate or severe anxiety. If we combine the mild, moderate and severe anxiety categories, two-thirds (65%) of the sample had some degree of anxiety. This number is troubling, especially in comparison with recent data from China (Lai et al., 2020) that found a much lower percentage (45%) of PHCs with mild, moderate or severe anxiety. Even the percentage of people with moderate and severe anxiety in our study was double the number reported in the Chinese study (12.3%).

In this study, female and young HCWs were more likely to experience moderate to severe anxiety compared to males and older HCWs. Similar findings have been reported among HCWs and the public (Ahmed et al., 2020; Wang et al., 2020). Huang and Zhao (2020) found that young HCWs reported higher anxiety than older ones. Likewise, Zhang and colleagues (2020) found that being female and being at risk of interacting with COVID-19 patients were associated with anxiety and depression.

Stress level was high among our participants, especially among females, and young HCWs. The mean score of 24 on PSS-10 in this study was high compared to several studies with different populations (E. H. Lee, 2012; Nordin & Nordin, 2013). The closest mean to our study was reported by A. M. Lee and colleagues (2007) who found that 1 year after the SARS outbreak in Hong Kong, HCWs who survived the virus had significantly higher scores on the PSS-10 (22.8) compared to non-HCWs (18.4). We also found that HCWs who worked with COVID-19 patients reported a higher level of stress compared to those who did not. Being on the frontline and dealing with positive or suspected COVID-19 patients and maintaining optimism while trying to save lives and protecting oneself and significant others from infection is undoubtedly stressful. Lai et al. (2020) found that HCWs who worked directly with COVID-19 patients in China were at risk of several psychiatric symptoms, including depression, anxiety and distress.

The overall psychological well-being among HCWs in this study was poor, with female and those who had cared for COVID-19 patients experiencing lower well-being. In particular, a cutoff value of <28 leads to labeling 86 HCWs with the possibility of having depression. Moreover, those who scored <28 also scored high on the anxiety scale.

In all three measures used in this study, females HCWs fared worse than males. They reported elevated levels of anxiety and stress and poor psychological well-being. Working closely with COVID-19 patients is worrying and has a detrimental effect on HCWs’ psychological health. In addition, on all three measures, older HCWs fared well compared to young ones. One potential interpretation of this is that older HCWs have experienced a great deal in their practice, and hence have developed better coping skills. HCWs who cared for COVID-19 patients reported a higher level of stress and poorer psychological well-being. W. Lu and associates (2020) found higher rates of fear, anxiety and depression among HCWs who worked in high COVID-19 risk areas compared to those who worked in areas with low risk of COVID-19 contact and compared to nonmedical hospital staff. Similarly, in one study (Zhang et al., 2020), compared to nonmedical staff, HCWs reported a higher prevalence of anxiety (13.0% vs 8.5%), insomnia (38.4% vs 30.5%) and depression (12.2% vs 9.5%).

During this international health crisis, the stress provoked by COVID-19 leads to a further proliferation of stress. That is, caring for COVID-19 patients, as a primary source of stress, begets new stressors associated with fear of infection, unfamiliar clinical roles, longer working hours and finding childcare when schools and daycares are closed.

An emotionally strong health care workforce is a critical component in the fight against the spread COVID-19 and healing its victims. In this study, we have only scratched the surface of the effects of COVID-19 on the HCWs who risk their lives to save others’ lives. This is eloquently illustrated by several physicians who wrote about their experience dealing with COVID-19. They stated that they have witnessed pain and suffering and faced many challenges throughout their professional careers, but they

never anticipated how practicing medicine in New York City at the front line of the COVID-19 pandemic would lead to the worst days of our careers. We have lost the intimate connection with our patients at their most vulnerable points; felt powerless in the face of the very real fear felt by patients, trainees, and our colleagues alike; and, worst of all, have been left unprotected. (Cunningham et al., 2020, p. 1)

Support for HCWs, especially those who display signs of trauma and stress, is critical as we go through this catastrophic global pandemic. Fear of contagion, stress, anxiety and concern for their well-being and significant others endanger the mental health of HCWs. Some mental health problems, as observed in previous disease outbreaks, might lead to maladaptive coping behaviors, including substance abuse and even suicide. A study among hospital employees in China conducted 3 years after the SARS outbreak found a positive relationship between exposure to the outbreak and alcohol abuse and dependence symptoms (Wu et al., 2009). There are many options for health care leaders to support and protect HCWs during this difficult time, such as implementing mindfulness and cognitive behavioral therapy intervention programs (Alikhani et al., 2020; Greenberg et al., 2020; Melnyk et al., 2020). In China, HCWs were provided with psychological intervention techniques including psychological assistance hotline and stress relief activities (Chen et al., 2020). Albott and colleagues (2020) developed a psychological intervention approach, based on the US Army’s Battle Buddies peer support model, called the Psychological Resilience Intervention. It consists of three levels. The first level of intervention provides peer support to all HCWs. The second level provides unit-level specific support through particular mental health consultants. The third level focuses on HCWs who experienced heightened level of stress and other mental health issues.

This study provided important results for future comparative studies among HCWs in other countries around the world. The results of this study are consistent with past results on the impacts of COVID-19 on the mental health of HCWs and extend those results based on data from the Arab world. However, despite its valuable contribution, it has some limitations. First, we relied on a convenience sample, which is not representative of HCWs in Oman. Therefore, the findings are not generalizable and should be interpreted with some caution. Second, the cross-national design of this study prohibits us from drawing conclusions about causality. Thus, more research is needed, especially longitudinal studies, to allow us to identify the important causes of psychiatric problems among HCWs. Finally, because we recruited our participants via WhatsApp, we could not calculate a response rate.

## Conclusion

To conclude, more work needs to be done to examine the psychological and social well-being of HCWs in other Arab countries. Since the COVID-19 pandemic has been in force for only a few months, the extent of its physical and emotional destruction has not been fully realized and documented.
